# Fermented Plant Extract-Loaded Collagen Scaffolds: Bioactive Hydrogels for Enhanced Wound Repair and Immune Modulation

**DOI:** 10.3390/gels12020129

**Published:** 2026-02-01

**Authors:** Lesly Katleya Usme-Duque, Miguel A. Medina-Morales, María I. León-Campos, Marisol Cruz-Requena, Leopoldo J. Ríos-González, Rebeca Betancourt-Galindo, Jesús A. Claudio-Rizo

**Affiliations:** 1 Agroenviromental Biotechnology Group, Laboratorio de Biotecnología Ambiental, Facultad de Ciencias Químicas, Universidad Autónoma de Coahuila, Blvd. V. Carranza s/n, República Oriente, Saltillo Coahuila 25280, Mexico; katleya_usme@uadec.edu.mx (L.K.U.-D.); marisol.cruz@uadec.edu.mx (M.C.-R.); leopoldo.rios@uadec.edu.mx (L.J.R.-G.); 2 Laboratorio de Materiales Avanzados, Facultad de Ciencias Químicas, Universidad Autónoma de Coahuila, Blvd. V. Carranza s/n, República Oriente, Saltillo Coahuila 25280, Mexico; ileanaleon@uadec.edu.mx; 3 Centro de Investigación en Química Aplicada, Enrique Reyna H. 140, San José de los Cerritos, Saltillo Coahuila 25294, Mexico; rebeca.betancourt@ciqa.edu.mx

**Keywords:** collagen hydrogels, *Flourensia cernua* extract, polyphenols, controlled release, wound healing, tissue regeneration

## Abstract

Fermented extracts of *Flourensia cernua* (*F. cernua*), enriched with bioactive polyphenols such as caffeic acid, apigenin, myricetin, and quercetin, exhibit strong potential to promote tissue regeneration. However, controlled delivery systems are required to enhance their bioavailability and therapeutic efficacy. In this study, *F. cernua* extracts (7–21 wt.%) were encapsulated in collagen hydrogels to develop bioactive matrices with sustained release properties. The hydrogel with 14 wt.% enabled sustained extract release from day 5 under physiological conditions and skin-mimicking pH (4.5). Increasing the extract concentration led to enhanced hydration behavior (>1400%) and crosslinking density (>45%), contributing to faster gelation. SEM analysis revealed fibrillar morphologies with amorphous globular domains whose prevalence increased with extract content and conferred improved thermal stability. Mechanical analysis indicated a decrease in matrix stiffness due to repulsive interactions between the extract components and the polymer network. Biodegradation studies showed slow hydrolytic and enzymatic degradation at skin pH in hydrogels containing 7 wt.% extract. All hydrogels demonstrated hemocompatibility, with no erythrocyte lysis. Moreover, hydrogels with 14 wt.% extract significantly enhanced the metabolic activity and proliferation of monocytes and fibroblasts, while 7 wt.% extract reduced TNF-α secretion, indicating anti-inflammatory potential. In vitro wound closure assays revealed 90% contraction within 10 days in fibroblast cultures exposed to 14 wt.% extract-loaded hydrogels. These results support the use of *F. cernua*-enriched collagen hydrogels as multifunctional scaffolds for wound healing and tissue regeneration.

## 1. Introduction

Semi-desert zones have a wide diversity of plants that represent a valuable source of bioactive compounds due to their adaptation to extreme environmental conditions. Such conditions promote the synthesis of secondary metabolites, including flavonoids and phenolic compounds [1,2,3]. Plant extracts obtained from semi-desert plants have shown great potential for biomedical applications, especially in the treatment of wounds and inflammatory diseases, by modulating key cellular processes such as cell proliferation, migration, and differentiation [4]. *F. cernua*, a plant native to semi-desert regions and used in traditional medicine to treat skin infections, wounds and respiratory diseases, has shown the presence of bioactive compounds such as caffeic acid, apigenin, myricetin, and quercetin. Its extracts have demonstrated anti-inflammatory, anti-proliferative and antioxidant properties, and have therefore been suggested as anti-inflammatory and anti-cancer agents [5,6,7,8].

Fermentation is an effective biotechnological strategy to improve the bioavailability and bioactivity of plant extracts by transforming their secondary metabolites through the action of selected microorganisms [9,10]. This process increases the stability and solubility of bioactive compounds and can generate metabolites with higher therapeutic efficacy and lower toxicity [11]. Thus, fermentation of *F. cernua* extracts is an effective strategy to optimize their bioactive profile, positioning it as a promising option in biomaterials engineering with biomedical applications, especially in the design of scaffolds and advanced materials for wound healing [12]. However, to ensure the biological efficacy of these fermented extracts, the use of suitable delivery vehicles is essential to guarantee their targeted release and bioactivity. In this context, several strategies have been proposed for the encapsulation of plant-derived compounds, including micro/nanoparticles, liposomes, and polymeric matrices [13,14]. Among them, hydrogels have emerged as versatile platforms for the incorporation and controlled release of phytochemicals due to their high water content, tunable porosity, and biocompatibility [15,16,17].

Hydrogels are highly hydrated three-dimensional (3D) polymeric networks, capable of absorbing large amounts of water without dissolving [18]. Collagen and polyurethane hydrogels have become of great interest due to their potential to combine bioactive and mechanical properties, as well as their capacity for therapeutic agent encapsulation [19]. Collagen, the main structural protein of the extracellular matrix, promotes cell adhesion, proliferation, and migration, while polyurethane provides mechanical strength, elasticity, and control over the biodegradability of the system [15,20,21]. The incorporation of bioactive agents in hydrogels allows the design of multifunctional platforms that, in addition to serving as structural support, promote tissue regeneration through the sustained release of therapeutic compounds [22].

Previous studies have shown that collagen and polyurethane-based hydrogels improve healing by modulating inflammation, promoting angiogenesis, and stimulating tissue regeneration. However, these systems often require the addition of bioactive agents to enhance their efficacy and achieve healing in chronic wounds [20,23,24,25]. The integration of fermented *F. cernua* extracts into collagen–polyurethane hydrogels represent an innovative and unexplored strategy in the field of biomaterials for wound healing. While both collagen matrices and plant-derived extracts have independently shown promise in tissue regeneration, no studies to date have reported the encapsulation of fermented *F. cernua* bioactives in this hydrogel system. This dual-functional approach not only leverages the antioxidant and anti-inflammatory properties of the fermented plant metabolites but also exploits the structural, mechanical, and tunable biodegradability of collagen–polyurethane hydrogels [10,26,27]. By enabling the sustained release of *F. cernua* bioactives within a supportive matrix, this system introduces a novel multifunctional scaffold capable of providing localized biochemical cues and physical support simultaneously addressing key limitations in current wound care materials and offering new avenues for immunomodulation and enhanced tissue regeneration.

This study focuses on the development and characterization of collagen hydrogels encapsulating fermented extracts of *F. cernua*, with the aim of evaluating the influence of extract content on structural features, physicochemical properties, and in vitro biological response. We propose that these hydrogels could act as advanced 3D bioactive scaffolds for wound healing, providing a moist environment that promotes tissue regeneration and immune modulation. In this study, collagen-based networks are primarily investigated in their hydrogel state, which represents the functional form relevant for swelling behavior, extract release, and biological performance. Xerogels correspond to the dried state of the same hydrogel networks and were employed exclusively for structural characterization, rather than as the intended application form.

## 2. Results and Discussion

### 2.1. Total Polyphenol Content and Antioxidant Activity of Fermented F. cernua Extract

The fermented extract of *F. cernua* exhibited a significant concentration of total phenolic compounds, measured at 36.84 ± 3.51 mg GAE per gram, and flavonoids, quantified at 28.69 ± 3.19 mg quercetin equivalents per gram. These phytochemicals contributed to the extract’s strong antioxidant potential, demonstrated by radical inhibition percentages of 80.7 ± 0.47% against DPPH and 94.3 ± 0.17% against ABTS radicals. High-performance liquid chromatography coupled with mass spectrometry (HPLC-MS) analysis revealed a rich profile of bioactive constituents, notably caffeic acid, apigenin, myricetin, and quercetin [12]. These compounds are well-recognized for their potent antioxidant, anti-inflammatory, and immunomodulatory properties, which are crucial in wound healing processes [12].

Following a pretreatment step involving ethanol removal by rotary evaporation, centrifugation, and hydrogen peroxide sterilization, a decrease in total phenolic content (4.7 ± 0.3 mg GAE/g) and flavonoids (3.65 ± 0.4 mg QE/g) was observed. However, antioxidant activity was largely preserved, indicating the stability of key bioactive molecules during processing, which is essential for their biological function when incorporated into hydrogels. The encapsulation of these phenolic and flavonoid compounds within hydrogel matrices enables controlled and sustained release, maintaining their bioavailability at the wound site [28]. This controlled delivery system leverages the known effects of caffeic acid, apigenin, myricetin, and quercetin in modulating inflammatory responses, promoting fibroblast proliferation, and scavenging reactive oxygen species [29,30,31,32]. Consequently, the synergy between the hydrogel platform and the phytochemical profile of *F. cernua* extract could enhance wound healing outcomes and immunomodulation by reducing inflammation and oxidative stress.

### 2.2. Phytochemical-Modulated Water Retention Capacity, Crosslinking, and Gelation in Collagen Hydrogels

**Figure 1:**
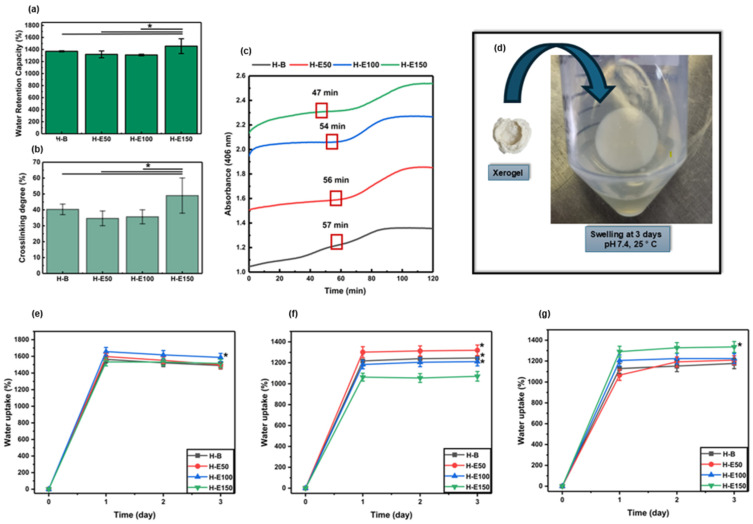
(**a**) Maximum water retention capacity (%) of hydrogels evaluated immediately after synthesis (native swelling behavior). (**b**) Crosslinking rate (%) and (**c**) polymerization kinetic curves of hydrogels with variable contents (0–21 wt.%) of *F. cernua* extract. (**d**) Representative photograph showing the rehydration and swelling recovery of a xerogel (H-E100) upon contact with 1× PBS at physiological pH (7.4). Swelling behavior of xerogels under different hydrolytic conditions after 72 h of immersion: (**e**) pH 4.5 (skin-related conditions), (**f**) pH 7.4 (physiological conditions), and (**g**) pH 8.5 (alkaline conditions associated with infected wound environments). * indicates statistically significant differences compared to the extract-free hydrogel matrix (H-B) (*p* < 0.05).

The maximum water retention capacity of the developed hydrogels after preparation is shown in Figure 1a. The water retention capacity values recorded are 1370 ± 8.63% for H-B, 1319 ± 56.2% for H-E50, 1309 ± 12.4% for H-E100, and 1456 ± 122% for H-E150. All formulations demonstrated water retention capacity above 1300%, classifying them as highly absorbent hydrogels. Such behavior is attributed to the abundance of hydrophilic functional groups (e.g., –OH, –COOH, –NH_2_) within the collagen network and the polar phytochemicals present in the *F. cernua* extract.

Statistical analysis reveals a significant increase in the water retention capacity of the hydrogel containing the highest concentration of *F. cernua* extract (H-E150) compared to both the control hydrogel (H-B) and those with lower extract content (H-E50 and H-E100) (*p* < 0.05). This result suggests that a critical concentration of phytochemicals may enhance the hydrogel’s water absorption capacity, likely due to the contribution of polar functional groups present in the bioactive compounds, which promote water retention and potentially modify the microstructure of the polymeric matrix.

The significant increase observed in H-E150 may be linked to the high content of bioactive compounds such as caffeic acid, quercetin, apigenin, and myricetin, previously identified by HPLC-MS in the extract. These molecules possess multiple hydroxyl and carboxyl groups capable of forming hydrogen bonds with water, thus enhancing the hydrogel’s water retention capacity. Moreover, their interaction with the polymeric matrix may induce microstructural changes, increasing porosity and water uptake capacity. From an application standpoint, this high water retention capacity ability is particularly advantageous for wound healing. Absorbent hydrogels are essential for managing wound exudate, maintaining a moist microenvironment, and supporting tissue regeneration. The elevated water retention also facilitates the controlled release of encapsulated phytochemicals, which may exert antioxidant and immunomodulatory effects at the wound site. Therefore, the enhanced water retention capacity of H-E150 not only reflects its physicochemical adaptation to biological environments but also its therapeutic potential in promoting efficient wound healing through both passive (fluid management) and active (bioactive compound delivery) mechanisms [33,34,35,36].

Figure 1b shows the crosslinking percentages of the developed hydrogels, indirectly determined using the ninhydrin assay by quantifying free amino groups. The recorded values are 40.34 ± 3.31% for H-B, 34.68 ± 4.61% for H-E50, 35.67 ± 4.40% for H-E100, and 49.02 ± 11.05% for H-E150. These results indicate that all formulations exhibit relatively crosslinking densities lower than 50%, which is characteristic of physically structured polymer networks. Such low-density networks enhance flexibility and favor high water retention capacity by allowing increased water diffusion through the matrix. However, this structural openness may reduce mechanical stability, limiting their applicability in contexts requiring high mechanical strength [37].

Notably, a statistically significant increase in crosslinking is observed in H-E150 compared to the control (H-B) and other extract-loaded matrices (*p* < 0.05). This behavior can be associated with the higher concentration of *F. cernua* extract. These molecules are rich in polar functional groups (–OH, –COOH, –NH_2_) that can form hydrogen bonds with the amino groups present in the collagen chains. As a result, a more homogeneous and physically crosslinked polymer network is generated, especially at higher extract concentrations [38]. In this context, the increase in reticulation refers predominantly to physical, non-covalent interactions between collagen chains and the polyphenol-rich extract, including hydrogen bonding, π–π interactions among aromatic moieties, and electrostatic interactions. These interactions are dynamic and reversible in nature and do not act as permanent covalent crosslinks restricting network expansion. Although higher extract concentrations increase the density of physical interactions within the network, these interactions are predominantly hydrophilic and reversible in nature. The polyphenol-rich extract introduces polar functional groups and disrupts collagen fibrillar packing, leading to enhanced osmotic swelling and increased free volume. Consequently, the swelling capacity increases despite higher interaction density, highlighting that physical reticulation in this system does not translate into effective elastic restriction of the network.

This crosslinking enhancement aligns with the extract’s previously characterized phytochemical composition, which exhibited high levels of total phenolic compounds and flavonoids, as well as strong antioxidant activity. Although the pretreatment process reduced the phenolic and flavonoid content, antioxidant capacity was retained, suggesting the persistence of chemically stable and biologically active components. These residual phytochemicals likely remain available for interaction within the hydrogel matrix and for biological activity post-application. The presence of these compounds contributes not only to structural modifications—such as increased crosslinking and water retention capacity—but also to the biomedical functionality of the hydrogels. Their inclusion could support sustained release of antioxidant and anti-inflammatory agents, critical for modulating the wound microenvironment. This dual function, combining structural adaptability with therapeutic efficacy, could enhance the hydrogel’s potential as an advanced wound dressing capable of reducing inflammation, promoting immune regulation, and accelerating tissue repair [39].

However, the simultaneous increase in water retention capacity and interaction density observed for H-E150 suggests a non-classical network behavior. In conventional polymeric networks, an increase in crosslinking density is typically associated with reduced water retention capacity. However, this relationship is not necessarily applicable to hybrid collagen-based systems covalently crosslinked with polyurethane. In the present system, the polyphenol-rich extract does not act as an additional covalent crosslinker, but rather as a structural modulator that interferes with collagen fibrillar packing. Through extensive hydrogen bonding with collagen chains, the extract promotes a less ordered, more amorphous network with increased free volume and a higher density of hydrophilic domains, which facilitates water uptake even at higher effective interaction densities.

The gelation kinetics of the developed hydrogels are presented in Figure 1c. The data show that increasing the concentration of *F. cernua* extract within the formulations progressively reduces the duration of the nucleation phase (47 min), indicating a faster onset of gel formation. In particular, the hydrogel containing 21 wt.% of extract (H-E150) exhibited a statistically significant acceleration in the nucleation stage compared to the control and lower extract-loaded matrices (*p* < 0.05). This enhancement suggests that specific phytochemicals in the extract could act as nucleation centers, promoting early-stage organization and polymer network formation through short-range physicochemical interactions.

This acceleration is likely mediated by hydrogen bonding and electrostatic interactions between the polar functional groups of phenolic compounds and the amino groups in collagen. These interactions favor early-stage physical crosslinking and contribute to the formation of more structured hydrogel matrix. Despite the rapid onset of gelation, the complete gelation process extended to approximately 1.8 h, which can be considered relatively slow. This prolonged timeframe is likely due to hydrophobic interactions between some extract components and the polar residues of collagen and polyurethane, which may interfere with the proper alignment and organization of fibrillar structures during the propagation phase [38].

Nonetheless, a stable gel state was achieved after 47 min, indicating the progression of intermolecular interactions and entanglements necessary for network formation. This controlled gelation process could be beneficial, as it promotes more uniform polymer chain organization, ultimately enhancing the hydrogel’s water absorption capacity. Indeed, these findings correlate with the high water retention capacity observed for H-E150 (1456 ± 122%), reinforcing the notion that an optimized level of phytochemical incorporation not only accelerates gel formation but also improves the functional properties of the hydrogel. From a biomedical perspective, these improvements are particularly relevant for wound dressing applications. The faster gelation enables efficient scaffold formation directly at the application site, while the enhanced water retention capacity ensures effective exudate absorption and maintenance of a moist healing environment. Moreover, the gradual release of bioactive phytochemicals from the structured network could provide sustained antioxidant and immunomodulatory effects, supporting tissue regeneration and inflammation control in situ [40,41].

Figure 1d illustrates the rehydration behavior of a representative xerogel (H-E100), showing its ability to swell upon contact with 1× PBS at physiological pH (7.4). The swelling behavior of the xerogels under different hydrolytic conditions is summarized in Figure 1e (pH 4.5, skin-related conditions), Figure 1f (physiological pH, 7.4), and Figure 1g (alkaline pH 8.5, associated with infected wound environments).

At acidic pH (4.5), the xerogels exhibited high swelling capacities, reaching values between 1450 and 1680% after 24 h of immersion. Notably, H-E100 showed statistically significant differences compared to the other formulations. This behavior can be attributed to enhanced protonation of polar functional groups present in collagen and the extract-derived phenolic compounds, which promotes electrostatic repulsion within the polymer network and facilitates water uptake despite the increased reticulation. Additionally, hydrogen bonding rearrangements under acidic conditions may contribute to a more expanded network conformation.

At physiological pH (7.4), swelling values after 24 h ranged from 1050 to 1320%, with statistically significant differences observed between H-B, H-E50, and H-E100 compared to H-E150. The reduced swelling capacity of H-E150 suggests that a high content of *F. cernua* extract limits water absorption at neutral pH. This effect is associated with the higher density of physical crosslinking induced by non-covalent interactions (hydrogen bonding, π–π stacking, and electrostatic interactions) between phenolic compounds and the polymeric matrix, which restricts chain mobility and reduces the availability of free volume for water penetration under neutral conditions.

Under alkaline conditions (pH 8.5), swelling capacities between 1040 and 1325% were observed after 24 h, with H-E150 displaying significantly higher swelling compared to the other formulations. At this pH, partial deprotonation of acidic groups and phenolic moieties enhances electrostatic repulsion within the network, counterbalancing the higher reticulation density and promoting increased water uptake. This indicates that, at alkaline pH, the extract-rich network becomes more responsive due to pH-triggered charge effects, leading to improved swelling performance.

Overall, these results demonstrate that swelling behavior is not solely governed by reticulation density but arises from a balance between network crosslinking and pH dependent physicochemical interactions. While increased extract content enhances reticulation through physical interactions, the ionization state of functional groups modulates chain mobility and osmotic forces, resulting in distinct swelling responses under acidic, neutral, and alkaline conditions.

### 2.3. Structural and Morphological Analysis Induced by F. cernua Extract in the Scaffold

**Figure 2:**
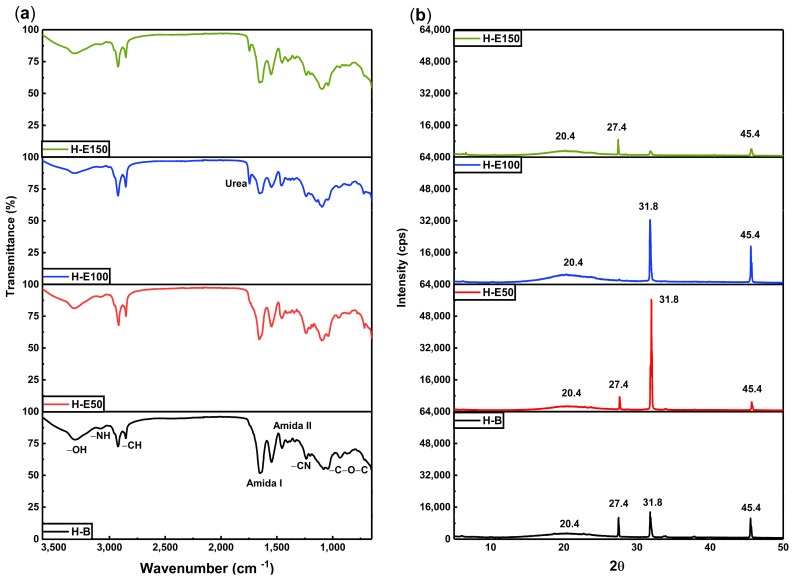
(**a**) Chemical structure by FTIR spectra and (**b**) XRD patterns for hydrogels with varying content of immobilized extracts (0–21 wt.%) of *F. cernua*.

The infrared spectra of hydrogels containing increasing concentrations of *F. cernua* phenolic extract are shown in Figure 2a. The FTIR analysis reveals distinctive absorption bands that confirm the coexistence and interaction of collagen, polyurethane, and phenolic compounds within the polymeric matrix.

In the 3500–3800 cm^−1^ region, broad bands corresponding to OH and NH stretching vibrations are evident. These groups are associated with both collagen and the phenolic compounds, particularly flavonoids and phenolic acids. A progressive increase in the intensity of these bands with higher extract content indicates an enrichment of hydrogen bonding interactions, suggesting short-range physicochemical crosslinking between the collagen network and phytochemicals. These interactions contribute to the improved water retention capacity and crosslinking behaviors observed previously, by reinforcing the hydrogel network through extensive hydrogen bonding interactions, while inducing structural rearrangements such as local amorphization and reorganization of the fibrillar structure of the collagen matrix.

The 2900–2700 cm^−1^ region shows persistent C–H stretching bands from the collagen backbone [42], confirming the structural integrity of the biopolymer across all formulations. Additionally, the sharp band at 1745 cm^−1^, assigned to the C=O stretching in urea groups (NH–CO–NH) formed during polyurethane crosslinking, becomes more intense in extract-loaded hydrogels, particularly in H-E150. This supports the hypothesis that phenolic compounds could act as facilitators of crosslinking, enhancing the density of the polymeric network through additional hydrogen bonding and possibly π–π stacking interactions. An increase in the intensity of the amide I (~1650 cm^−1^) and amide II (~1540 cm^−1^) bands is also observed with increasing extract concentration. This enhancement suggests structural stabilization of the collagen chains through phenolic interactions, likely via hydrogen bonds and secondary coordination with polar moieties in the extract. Such molecular stabilization is crucial for hydrogel performance in biological environments, where matrix disintegration must be carefully controlled [43].

The spectral region between 1300 and 1200 cm^−1^ displays signals attributed to C=N and C–N stretching vibrations, indicative of chemical integration of the phenolic compounds into the matrix. These bands highlight the successful functionalization of the hydrogel and confirm that phenolic molecules are not merely adsorbed but are embedded and potentially immobilized within the polymer network. This is further supported by the increase in the C–O–C band at ~1100 cm^−1^, characteristic of glycosidic linkages present in polyphenolic glycosides, which also intensify proportionally with extract content. From a therapeutic standpoint, this chemical configuration could allow the sustained release of bioactive compounds with antioxidant and immunomodulatory activity of *F. cernua*, which are crucial for managing oxidative stress and inflammation during wound healing [44]. Furthermore, the molecular anchoring of phenolic compounds could prevent premature leaching, ensuring prolonged in situ activity that supports tissue regeneration [45].

Figure 2b displays the X-ray diffraction (XRD) patterns, the hydrogel without extract (H-B) exhibits a broad amorphous halo centered at 20.4°, which is attributed to the flexible and disordered nature of the polyurethane crosslinking regions. In contrast, the organized fibrillar structure of collagen is evidenced by distinct crystalline peaks at 27.4°, 31.8°, and 45.4°, indicating a semi-crystalline architecture in the scaffold [46,47]. Upon the incorporation of the *F. cernua* extract, significant structural alterations are observed. At extract concentrations of 7 wt.% and 14 wt.% (H-E50 and H-E100), the diffraction peak at 31.8°, associated with collagen fibril alignment, becomes more pronounced. This suggests that moderate levels of phytochemicals enhance molecular ordering and potentially reinforce hydrogen bonding within the collagen network, promoting local structural regularity. Concurrently, the amorphous halo at 20.4° increases in intensity with higher extract content, indicating that phytochemicals interact preferentially with the polyurethane domains, potentially disrupting local order and increasing matrix heterogeneity. Interestingly, at the highest extract concentration (21 wt.%, H-E150), a marked decrease in the intensity of the collagen-related crystalline peaks is observed, implying that excessive phytochemical content disrupts collagen fibrillogenesis or impairs molecular packing within the scaffold. This phenomenon could result from oversaturation of the matrix with bioactive compounds, leading to steric hindrance or excessive hydrogen bonding of crosslinking that inhibits organized assembly of collagen fibers.

These structural modifications correlate well with previous results from gelation kinetics and crosslinking analyses. The extract-induced modulation of the hydrogel’s crystalline–amorphous balance reflects a delicate interplay between the bioactive compounds and the polymeric components of the scaffold. Such tunable structural organization is critical for applications in wound healing and immunomodulation, as it influences both the mechanical integrity and the controlled release behavior of the scaffold [41,48]. The observed semi-crystalline reorganization and amorphous domain expansion could enhance water retention and bioactive diffusion, providing a multifunctional environment favorable for tissue regeneration and inflammatory control [49].

This loss of long-range collagen order at high extract concentrations supports the notion that increased physical crosslinking does not necessarily translate into higher mechanical stiffness, but rather into a more heterogeneous and flexible polymer network.

**Figure 3:**
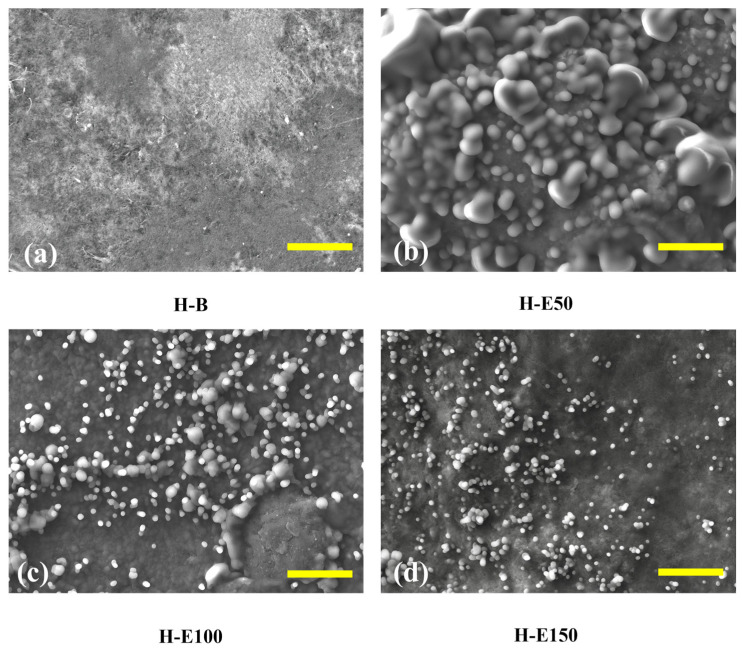
Surface structure of hydrogels with varying content of immobilized extracts (0–21 wt.%) of *F. cernua* by SEM (scale bar = 10 µm): (**a**) H-B, (**b**) H-E50, (**c**) H-E100 and (**d**) H-E150.

Figure 3 presents SEM images of collagen-based xerogels obtained by controlled dehydration of the corresponding hydrogels. These images are used for a comparative evaluation of extract-induced structural organization. It should be noted that SEM analysis was performed under high-vacuum conditions and therefore provides an indirect, qualitative assessment of network morphology, which is employed here to identify relative morphological trends among the different formulations. In Figure 3a (H-B), the control sample without extract displays a well-organized fibrous collagen network with interconnected porosity, which suggests a network architecture potentially favorable for water retention and nutrient diffusion in the hydrated state. This porous structure provides a suitable scaffold for tissue ingrowth and cellular infiltration [50]. In Figure 3b (H-E50), corresponding to hydrogels with 7 wt.% extract, globular domains appear sparsely distributed across the surface. These globules likely correspond to agglomerates of hydroxyl and carboxylate-rich phenolic compounds that became entrapped within the collagen fibrils. Their uneven distribution suggests incomplete integration, promoting localized physical entanglement and increasing the apparent crystallinity of the collagen fibers. These domains could function as isolated reservoirs of bioactive molecules; however, their release is likely less homogeneous and more erratic, potentially limiting controlled therapeutic efficacy [51].

In contrast, Figure 3c (H-E100), at 14 wt.% extract, reveals a more uniform and denser distribution of finely dispersed globular particles across the fibrillar surface. This indicates a more efficient immobilization of the phenolic compounds within the polymeric matrix, likely due to enhanced hydrogen bonding and π–π stacking interactions. The decrease in particle size and improved dispersion are consistent with increased molecular repulsion between hydroxyl groups and partial saturation of hydrogen bonds, leading to moderate steric effects within the network. This configuration suggests an amorphous-dominated organization associated with enhanced physicochemical interactions, which enhances the hydrogel’s water retention capacity, while could enable a more gradual and sustained release of phytochemicals [41].

At the highest extract content, Figure 3d (H-E150), the surface morphology becomes more heterogeneous, with smaller, irregular granules of phenolic material embedded in the matrix. This suggests partial saturation of the hydrogel network, and the formation of extract-rich microdomains, indicative of microstructural heterogeneity rather than macroscopic phase separation. Despite the reduced uniformity, these smaller aggregates could facilitate more rapid self-assembly and stronger non-covalent crosslinking through dense hydrogen bonding and hydrophobic interactions.

This structural arrangement correlates with the previously observed increases in water retention capacity and gelation rate, underscoring the extract’s dual role in modulating both microstructure and functional performance.

These microstructural transitions—from localized agglomeration to uniform dispersion and finally to saturation-induced domain formation—highlight the tunability of the hydrogel system through phytochemical concentration of *F. cernua*. Such control is crucial for optimizing scaffold performance in wound healing applications, where both sustained bioactive release and structural adaptability to dynamic biological environments are essential for effective immunomodulation and tissue regeneration [52].

Although BET surface area analysis was not conducted in this study, previous reports on collagen–polyurethane matrices incorporating polysaccharides such as chitosan have demonstrated specific surface areas of approximately 75.8 m^2^ g^−1^, pore volumes around 0.07 cm^3^ g^−1^, and average pore radii of about 1.9 nm after dehydration [53]. These parameters indicate a mesoporous structure that facilitates water transport and molecular diffusion. Considering the structural similarities and the comparable physical crosslinking interactions present in the current systems, the xerogels investigated here are expected to exhibit porosity characteristics within a similar range, which is consistent with the high swelling capacities and interconnected microstructural features observed by SEM.

### 2.4. Degradation and Viscoelastic Properties of F. cernua-Loaded Scaffolds

**Figure 4:**
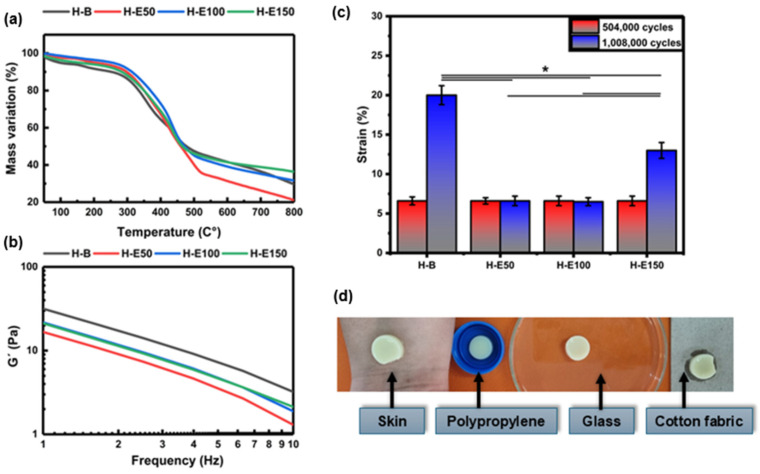
(**a**) Thermal degradation profiles obtained by thermogravimetric analysis (TGA) and (**b**) variation in storage modulus (G) as a function of frequency determined by oscillatory rheometry for hydrogels containing different amounts (0–21 wt.%) of *F. cernua* extract. (**c**) Fatigue behavior of the hydrogels evaluated after 504,000 and 1,008,000 loading cycles, showing the deformation response as a function of extract content. (**d**) Representative photographs illustrating the qualitative adhesion behavior of a hydrogel containing *F. cernua* extract (H-E100) on different substrates, including human skin, polypropylene, glass, and cotton fabric. Images were acquired with the hydrogel positioned in an inverted configuration (against gravity), demonstrating stable adhesion without detachment. * indicates statistically significant differences among the compared groups (*p* < 0.05).

Thermal stability of the collagen scaffolds encapsulating fermented *F. cernua* extracts was evaluated TGA, as shown in Figure 4a. All thermograms exhibit three distinct regions of mass loss. The first stage, occurring between 40 and 160 °C, corresponds to the evaporation of water and low molecular weight volatile compounds such as organic acids, alcohols, and terpenes derived from the phenolic extract. A mass loss of approximately 6% is observed in the control hydrogel (H-B), while extract-loaded scaffolds (H-E50, H-E100, and H-E150) display lower losses of 3–4%. Although the differences are not statistically significant, the presence of encapsulated extract appears to reduce the volatilization of labile compounds due to their stabilization within globular domains formed during scaffold formation. The second region, between 330 and 500 °C, is attributed to the endothermic degradation of the phenolic compounds, collagen, and polyurethane chains. Here, mass losses range from 53 to 57% for H-B, H-E100, and H-E150, indicating comparable thermal decomposition. In contrast, H-E50 exhibits a notably higher mass loss of 63%, which may be attributed to the presence of larger globular aggregates with greater surface area, increasing susceptibility to thermal degradation due to repulsive interactions among hydroxyl-rich phytochemical domains.

In the third stage (550–800 °C), corresponding to the exothermic decomposition of residual organic matter, ash formation is generated as a thermal degradation byproduct. The scaffold with the highest extract content (H-E150) retains the greatest residual mass (42%), suggesting that the smaller, more homogeneously dispersed globular aggregates occluded within the fibrillar network confer enhanced thermal resistance due to increased physicochemical crosslinking. Moreover, it has been reported that the fermented extract of *F. cernua* is rich in inorganic species such as magnesium, calcium, and silicon, which contribute to the increased ash formation observed during thermal degradation [12]. Conversely, H-E50 shows the lowest residual mass (20%), supporting the hypothesis that scaffold morphology—specifically, globular aggregate size and distribution—plays a critical role in regulating intermolecular interactions between phytochemicals and the collagen–polyurethane matrix. These findings reinforce the multifunctional potential of *F. cernua*-loaded scaffolds for wound healing applications, where thermal stability, structural resilience, and controlled bioactive retention are essential for promoting sustained antioxidant and immunomodulatory responses during tissue repair [54].

Figure 4b displays the frequency-dependent storage modulus (G′) of the hydrogels. G′ serves as a fundamental indicator of the hydrogel’s mechanical stiffness and its ability to recover shape after deformation. The control hydrogel (H-B) exhibits the highest G′ (32 Pa at 1 Hz), denoting a more elastic and structurally ordered matrix, consistent with its fibrillar and semi-crystalline morphology observed via SEM and XRD. As the concentration of immobilized phenolic extract from *F. cernua* increases (H-E50 to H-E150), a progressive decrease in G′ is observed. In physically crosslinked collagen-based hydrogels, an increase in interaction density driven by hydrogen bonding may coexist with a reduction in storage modulus when these interactions disrupt fibrillar alignment and increase amorphous character, as observed in the present system. This reduction reflects a softening of the matrix likely due to the disruption of the highly organized collagen network, as evidenced by FTIR and SEM, where the formation of more amorphous and globular domains was identified. Particularly in H-E50 (16 Pa at 1 Hz), the larger and irregular phytochemical agglomerates interfere with the native fibrillar structure, reducing mechanical coherence.

At higher extract concentrations (H-E100 and H-E150) (23 Pa at 1 Hz), although the modulus remains lower than that of H-B, improved homogeneity and smaller, more evenly distributed globules help partially restore mechanical stability. These extract-rich scaffolds thus demonstrate an optimized balance between reduced rigidity and enhanced adaptability [55]. This tailored viscoelastic behavior is critical for biomedical applications, especially wound healing, where softer matrices can promote better cellular infiltration and tissue integration, while retaining sufficient cohesion to withstand physiological stresses [56]. Additionally, the controlled decrease in mechanical stiffness correlates with increased amorphous character and improved water retention capacity, which together favor the regulated diffusion of bioactives [57]. Although the extract-containing hydrogels exhibit higher interaction density, the observed decrease in storage modulus indicates that increased physical reticulation does not necessarily lead to enhanced rigidity in collagen-based networks. The polyphenol-rich extract interferes with collagen fibrillar alignment, promoting amorphous domains and repulsive interactions that increase network heterogeneity and local chain mobility. As a result, the effective load-bearing capacity of the fibrillar network is reduced, leading to lower macroscopic stiffness despite increased secondary interactions.

Figure 4c shows the fatigue behavior of the hydrogels subjected to 504,000 and 1,008,000 loading cycles. After 504,000 cycles, all hydrogel formulations exhibited a similar deformation of approximately 6.6%, with no statistically significant differences among samples, indicating comparable short-term resistance to cyclic mechanical stress.

When the number of fatigue cycles was doubled to 1,008,000, clear differences in deformation behavior emerged depending on the extract content. The extract-free hydrogel (H-B) exhibited a pronounced increase in deformation, reaching approximately 21%, indicating progressive mechanical damage and reduced fatigue resistance. In contrast, hydrogels containing intermediate amounts of *F. cernua* extract (H-E50 and H-E100) maintained a low deformation level of approximately 6.6%, demonstrating enhanced resistance to long-term cyclic loading. The hydrogel with the highest extract content (H-E150) showed an intermediate deformation of approximately 13%.

Statistically significant differences in deformation resistance were observed between extract-containing hydrogels and the collagen–polyurethane matrix without extract. The improved fatigue resistance of H-E50 and H-E100 can be attributed to the formation of a physically reinforced network arising from non-covalent interactions between extract-derived phenolic compounds and the polymer matrix, including hydrogen bonding, π–π interactions, and electrostatic interactions. These reversible interactions act as dynamic sacrificial bonds, which dissipate mechanical energy during cyclic loading and delay the accumulation of irreversible damage.

At higher extract contents, however, excessive physical interactions may lead to increased network heterogeneity and localized stress concentration, resulting in a partial loss of fatigue resistance, as observed for H-E150. This suggests that while moderate extract incorporation enhances mechanical resilience through dynamic network reinforcement, excessively high extract loading can compromise fatigue performance by reducing structural uniformity and effective load distribution.

In addition to the structural and mechanical characterization, the adhesive behavior of the hydrogels was qualitatively evaluated to assess their ability to establish stable contact with different surfaces. Figure 4d presents representative photographs of a hydrogel formulation containing *F. cernua* extract (H-E100) adhered to various substrates, including human skin, polypropylene, glass, and cotton fabric.

The hydrogel was placed in an inverted configuration, with the material positioned against gravity on each substrate. Under these conditions, the hydrogel remained firmly attached without detachment, demonstrating good interfacial adhesion and mechanical stability across both biological and synthetic surfaces.

This adhesion behavior is consistent with the fibrillar microstructure observed by SEM and with the chemical composition of the hydrogel network, which contains multiple functional groups such as hydroxyl, carboxyl, amide, and phenolic moieties. These groups enable a combination of hydrogen bonding, electrostatic interactions, and surface wetting effects, promoting favorable interactions with hydrated biological tissues as well as polar and non-polar substrates.

Although quantitative adhesion measurements were not performed, the observed qualitative adhesion, together with the enhanced fatigue resistance of extract-containing hydrogels, supports their potential suitability for applications requiring conformal contact and mechanical stability under dynamic biological conditions.

**Figure 5:**
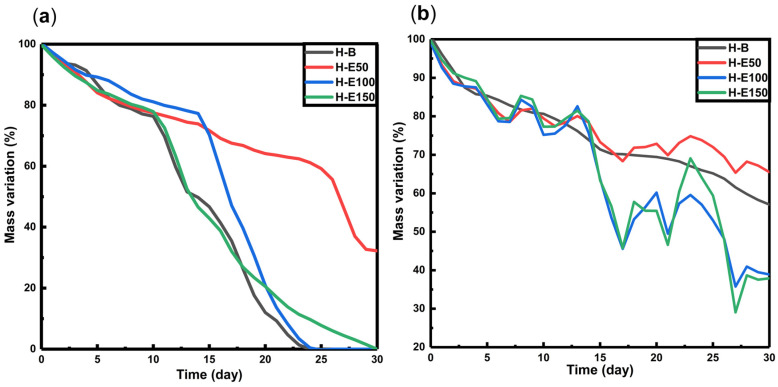
Degradation profiles of hydrogels with *F. cernua* extracts in contact with (**a**) type I collagenase (14 U per scaffold), and (**b**) pH: 4.5.

The degradation behavior of hydrogels loaded with fermented *F. cernua* extract was investigated under proteolytic conditions (collagenase type I, simulating enzymatic tissue remodeling) and hydrolytic conditions at skin pH (4.5), mimicking the acidic microenvironment of chronic wounds [58,59,60]. The mass loss profiles over time are presented in Figure 5.

Under proteolytic conditions (Figure 5a), all scaffolds exhibit a gradual mass loss of approximately 35–40% between days 15 and 18, with no significant differences observed across varying extract contents. However, by day 30, residual masses of 0% are found in scaffolds H-B, H-E100, and H-E150, while scaffold H-E50 retains 30% of its original mass. These results suggest that high concentrations of fermented *F. cernua* extract do not compromise the inherent biodegradability of the collagen network. This effect is likely promoted by the more uniform distribution of small globular extract domains on the scaffold surface, which do not hinder enzymatic access. In contrast, the increased crystallinity and presence of larger surface globular domains in H-E50 appear to reduce enzymatic susceptibility, potentially due to steric hindrance or lower enzymatic affinity to compacted regions, thus delaying proteolytic degradation.

In hydrolytic conditions at pH 4.5 (Figure 5b), all scaffolds show an initial mass loss of around 30% by day 15. Subsequently, swelling–shrinkage dynamics are observed, resulting in residual masses at day 30 of 58% for H-B, 67% for H-E50, 38% for H-E100, and 36% for H-E150. These data reveal significant differences in hydrolytic degradation profiles based on the amount of immobilized plant extract. The presence of *F. cernua* phytochemicals, capable of forming hydrogen bonds, appears to promote hydrolytic breakdown by disrupting the hybrid fibrillar–globular matrix structure, particularly in amorphous surface regions. Interestingly, scaffolds with larger globular domains (H-E50) exhibited slower degradation, likely due to partial entrapment of collagen fibers within phytochemical-rich microenvironments, reducing matrix hydration and accessibility.

These results demonstrate that the incorporation of fermented *F. cernua* extract enables fine-tailoring of the scaffold’s degradation behavior under physiologically relevant conditions, without compromising the native biodegradability of collagen. The presence of globular domains enriched with phytochemicals modulates the degradation kinetics, supporting the controlled release of bioactive compounds. This feature is particularly valuable for wound healing applications, where a scaffold must degrade in synchrony with tissue regeneration, allowing sustained delivery of antioxidant and immunomodulatory agents that support inflammation resolution and promote cellular repair [59,60].

### 2.5. Phytochemical Release Behavior Under Wound-Mimicking Conditions

**Figure 6:**
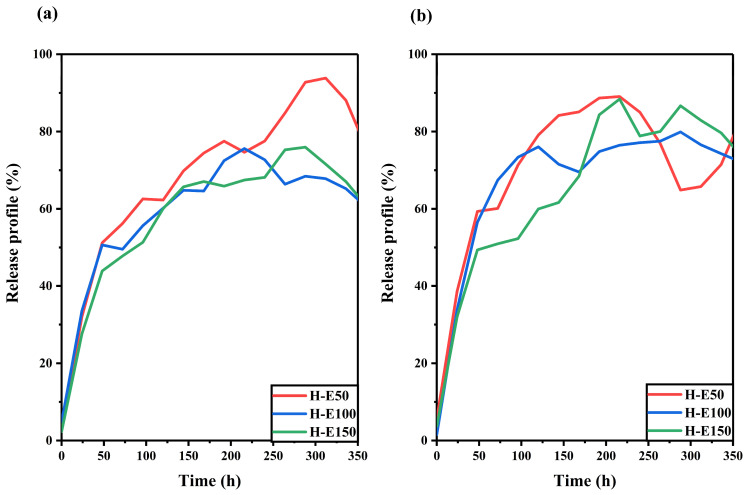
Release profiles of *F. cernua* phytochemicals from collagen scaffolds under (**a**) skin pH (4.5) and (**b**) physiological pH (7.4) conditions over time.

The release behavior of the *F. cernua* extract encapsulated within the collagen scaffold was studied under skin pH and physiological pH conditions, as shown in Figure 6.

At skin pH (Figure 6a), all scaffolds exhibit a linear release over time up to approximately 220 h (9 days). The profiles also reveal a progressive decline in release associated with the extract over the entire monitoring period (350 h, 15 days), suggesting a multidose-type release driven by hydrogel swelling [61]. Among the formulations, H-E50 shows the highest release (92%), indicating a greater release of phytochemicals. This behavior is attributed to its unique semicrystalline fibrillar–globular morphology, which facilitates water uptake and diffusion pathways, while also slowing hydrolytic degradation—allowing more sustained release. In contrast, the H-E100 and H-E150 scaffolds, which exhibit higher physical and chemical crosslinking due to the presence of the phytochemicals, show reduced extract release (62–73%). This is likely a result of increased water retention and stronger entrapment of bioactives within the denser matrix.

At physiological pH (Figure 6b), all scaffolds demonstrate a linear release pattern up to 225 h (approximately 9 days), followed by divergent release dynamics depending on the formulation. H-E50 exhibits a slight reabsorption of released extract, attributed to reversible swelling–diffusion phenomena. The H-E100 scaffold maintaines a sustained release up to 320 h (13 days), indicating a favorable balance between matrix cohesion and controlled release. Meanwhile, H-E150 shows a multidose release profile, likely driven by the interplay between amorphous surfaces and globular domains that modulate the diffusion of encapsulated phytochemicals. Interestingly, under physiological pH, the scaffold with the highest extract content (H-E150) achieves the greatest release after 240 h (10 days) (86%), which could be linked to its enhanced hydrophilicity and the disruption of polymeric networks by the extract’s globular phytochemical aggregates.

These release dynamics suggest that the tailored architecture of the hybrid scaffolds enables a temporally controlled delivery of phytochemicals, which is essential for supporting distinct phases of wound healing [61,62]. The early-phase sustained release can help modulate oxidative stress and pro-inflammatory signals, while the prolonged diffusion from more densely crosslinked matrices may contribute to later stages of tissue regeneration by maintaining a bioactive microenvironment [63,64]. The capacity to fine-tune release profiles based on scaffold composition and structure could position these systems as promising tools for guiding immunomodulatory responses and accelerating tissue repair in chronic and acute wound scenarios.

### 2.6. Modulation of Metabolic Activity and Proliferation by F. Cernua-Loaded Hydrogels in Key Wound Healing Cells

**Figure 7:**
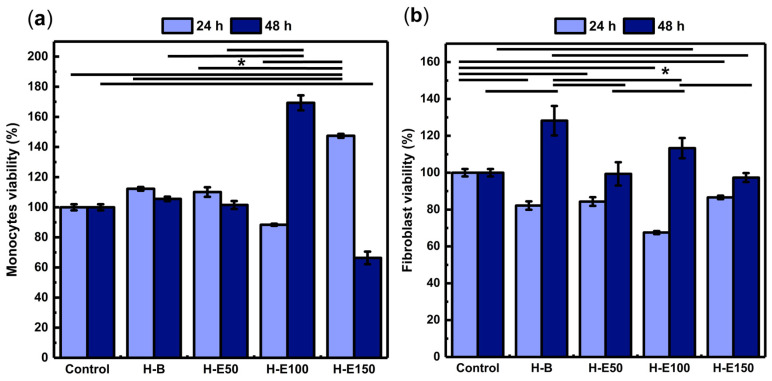
Assessment of the metabolic activity of key wound healing cell lines in response to hydrogels containing varying concentrations (0–21 wt.%) of immobilized *F. cernua* extract: (**a**) human monocytes and (**b**) porcine dermis fibroblasts. * indicates statistically significant differences among the compared groups (*p* < 0.05).

The influence of *F. cernua* extract content on the biological response of the scaffolds was first evaluated through the metabolic activity of key cell types involved in wound healing. Figure 7 shows the viability of animal cell lines after 24 and 48 h of incubation.

Monocytes play a pivotal role in orchestrating inflammatory resolution, fibrin remodeling, and neovascularization [65]. As shown in Figure 7a, all scaffolds exhibit viability above 70% at 24 h, indicating no cytotoxic effects. Notably, the H-E150 scaffold reaches the highest viability (146%), with statistically significant differences compared to the control and other groups. This enhanced early metabolic activity is likely linked to the scaffold’s fibrillar-globular microstructure, which facilitates localized extract retention, gradual release, and effective intracellular delivery of *F. cernua* phytochemicals. Such architecture supports sustained bioavailability within the pericellular environment, favoring early immunomodulatory activation.

At 48 h, all formulations—except H-E150—maintain viability above 80%, confirming long-term compatibility. However, H-E150 exhibites a moderate decline in metabolic activity (68%), suggesting a slight cytotoxic effect (ISO 10993-5). This behavior could be attributed to the fact that at moderate concentrations, phenols can act as antioxidants, protecting cells from oxidative damage and promoting viability; however, at high concentrations, phenolic compounds can become prooxidants, generating excessive oxidative stress that causes cell damage and reduces viability [66]. In contrast, the scaffold with 14 wt.% extract (H-E100) showes the highest viability at this timepoint (169%), significantly surpassing the control and other groups. This behavior can be attributed to a balanced scaffold structure that optimizes both the gradual release and spatial presentation of bioactives, enhancing monocyte metabolic performance. Collectively, these findings emphasize how scaffold morphology, extract content, and delivery profiles converge to modulate immune cell responses—an essential feature for promoting inflammation resolution and tissue repair in wound healing applications [67].

Fibroblasts are connective tissue cells that regulate extracellular matrix synthesis, playing a key role in healing, tissue remodeling, and intercellular signaling [68,69]. As shown in Figure 7b, after 24 h of culture, fibroblasts grown on the extract-containing scaffolds exhibit viability values ranging from 68% to 83%, indicating the absence or only mild cytotoxic effects (ISO 10993-5). Significant differences are observed compared to cells grown in the control (1× PBS), suggesting that the phytochemicals from *F. cernua* do not promote early-stage fibroblast metabolic activation. However, after 48 h, all scaffolds support enhanced metabolic activity, with the extract-free hydrogel (H-B) yielding the highest viability (128%), which is attributed to its fibrillar morphology devoid of globular regions—favoring cell adhesion and spreading once cells have adapted to the microenvironment. Among the extract-loaded scaffolds, H-E100 (14 wt.%) achieves a viability of 112%, significantly higher than H-E50 and H-E150. This behavior mirrors the trends observed in monocytes, where the intermediate extract concentration provided a favorable balance between scaffold structure and bioactive molecule availability. The globular-fibrillar morphology of H-E100 likely facilitates gradual phytochemical availability at the cellular interface while preserving a biocompatible environment. These results underscore the importance of matrix architecture and phytochemical dose in modulating fibroblast behavior—critical for supporting matrix deposition and re-epithelialization during wound repair [68,69].

**Figure 8:**
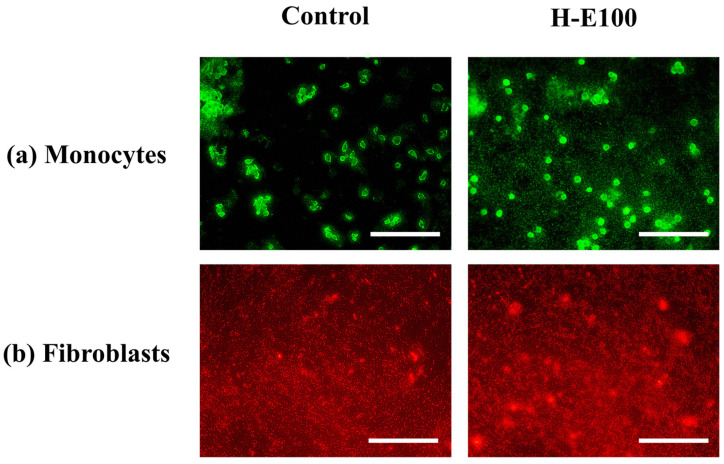
Fluorescence-based evaluation of cell proliferation in key cell lines involved in wound healing and immunomodulation after exposure to the H-E100 hydrogel (containing 14 wt.% immobilized *F. cernua* extract). Images were captured using a 40× objective (scale bar = 200 μm). (**a**) Human monocytes stained with fluorescein, indicating viable and proliferating immune cells. (**b**) Porcine dermis fibroblasts stained with rhodamine B, revealing dense, proliferative fibroblast populations.

For the evaluation of cell proliferation, only the H-E100 hydrogel was selected, as it demonstrated the most favorable combination of physicochemical characteristics and biological performance in terms of cell viability across the tested cell lines. The fluorescence microscopy images using fluorescein a membrane-permeant vital dye that accumulates in viable cells with intact membranes [70] for monocytes are shown in Figure 8a.

These images reveal abundant green-stained monocyte populations following exposure to the H-E100 hydrogel. The intense fluorescence indicates high cell viability and proliferation, highlighting the hydrogel’s ability to support immune cell expansion. This is likely due to the interaction between the scaffold’s microarchitecture and the bioactive phytochemicals—such as flavonols, flavones, methoxyflavones, and hydroxybenzoic acids—that promote cell survival and metabolic stimulation, as discussed previously [71].

Similarly, Figure 8b presents fluorescence images for fibroblats stained with rhodamine B (a dye that preferentially accumulates in the cytoplasm of live cells due to its affinity for mitochondrial and intracellular proteins [72]. The observed dense red-stained fibroblast populations in the presence of H-E100 hydrogel confirm an active proliferation process. These findings are consistent with the metabolic activity data reported earlier and suggest that the intermediate extract concentration and favorable scaffold morphology not only preserve fibroblast viability but also stimulate proliferation after adaptation. Together, these results reinforce the notion that the phytochemical-rich environment created by the fermented extract and its sustained interaction with the hydrogel matrix can effectively enhance immune and dermal cell activity—crucial features for promoting wound healing and tissue regeneration [41,73].

### 2.7. Systemic and Cellular Response: Hemocompatibility, Cytokine Secretion, and Tissue Regeneration

The safety of applying the scaffolds loaded with fermented *F. cernua* extract was assessed by measuring their hemocompatibility using a hemolysis assay with human erythrocytes. The hemolysis test quantifies the amount of hemoglobin released upon disruption of the red blood cell membrane. According to the regulation NOM-241-SSA1-2021 for medical devices, hemolysis values between 0% and 2% are considered indicative of low or negligible hemolytic potential. As shown in Table 1, all hydrogel formulations containing immobilized *F. cernua* extracts fall within this safe range (0–1.4%), confirming their hemocompatibility. These results suggest that the fibrillar collagen scaffold effectively encapsulates and gradually releases the phytochemicals without inducing osmotic stress or compromising red blood cell integrit [74]. Moreover, the superficial globule size associated with extract content does not significantly impact erythrocyte lysis, highlighting the biocompatible nature of the scaffold surface.

Previous observations (unpublished data) revealed that both fermented and macerated extracts of *F. cernua* exhibited high hemolytic activity when applied directly. This behavior is consistent with the dual nature of phenolic compounds, which can either stabilize or disrupt cellular membranes depending on their concentration and specific chemical structure [75,76]. However, when these phytochemicals are encapsulated within hydrogels, their release becomes gradual, allowing modulation of their bioavailability and interaction with eitrocytes [77]. This encapsulation significantly reduces direct contact with erythrocytes, contributing to the formation of hemocompatible biomaterial surfaces [78].

Together with the previously demonstrated metabolic stimulation and proliferative effects on immune and dermal cells, these findings underscore the potential of these scaffolds for wound healing applications. Moreover, the lack of hemolysis suggests potential for these materials to support hemostasis-related events, such as platelet adhesion and activation—key processes in wound healing. The controlled interaction between the phytochemicals and the hydrogel matrix ensures both cellular activation and blood compatibility—key attributes for developing advanced wound care systems with immunomodulatory benefits [71].

The immunomodulatory potential of the scaffolds was evaluated by monitoring the signaling of TNF-α, a key cytokine involved in inflammation. TNF-α acts as an early-stage immune mediator and is well known for its pro-inflammatory properties. It plays a critical role in tissue repair by promoting the activation of fibroblasts, macrophages, and keratinocytes, as well as contributing to extracellular matrix remodeling [79].

The results of TNF-α secretion by human monocytes after 48 h of contact with the hydrogels are shown in Table 1. The levels of TNF-α increase proportionally with the concentration of extract encapsulated in the hydrogel, with measured values of 0 pg/mL (H-B), 7.2 pg/mL (H-E50), 14.2 pg/mL (H-E100), and 34.2 pg/mL (H-E150). Statistically significant differences were observed in the H-E150 group compared to the other scaffolds. These trends suggest that *F. cernua* extracts, when encapsulated in the hydrogel matrix, induce a moderate, dose-dependent inflammatory response without evidence of hemolytic damage.

The increased cytokine secretion could be associated with surface features such as scaffold decreased crystallinity and topographical roughness induced by the globular morphology with higher crosslinking, both of which influence protein adsorption and cell activation [80]. It is noteworthy that baseline plasma levels of TNF-α in healthy individuals typically range between 0 and 20 pg/mL, depending on physiological conditions and assay sensitivity [81]. Accordingly, TNF-α levels induced by H-E50 and H-E100 remain within or just above the physiological range, indicating an activated but non-pathological immune response. In contrast, the H-E150 scaffold induces levels exceeding this threshold, suggesting a stronger immunostimulatory effect [82].

The inflammatory response is a critical phase of wound healing; however, excessive TNF-α levels may be detrimental, as they are linked to chronic inflammation and delayed tissue repair [83]. Therefore, the H-E150 hydrogel could be better suited for chronic or infected wounds, where a stronger pro-inflammatory environment is advantageous [83]. In contrast, H-E50 and H-E100 exhibit more moderate increases in TNF-α, likely supporting a more balanced transition between inflammation, cellular proliferation, and tissue remodeling [80]. These results demonstrate that incorporating plant extracts into hydrogels not only alters their physicochemical and structural properties but also directly modulates immune responses—an aspect of great relevance for biomedical applications such as tissue engineering and wound healing therapies [84].

**Figure 9:**
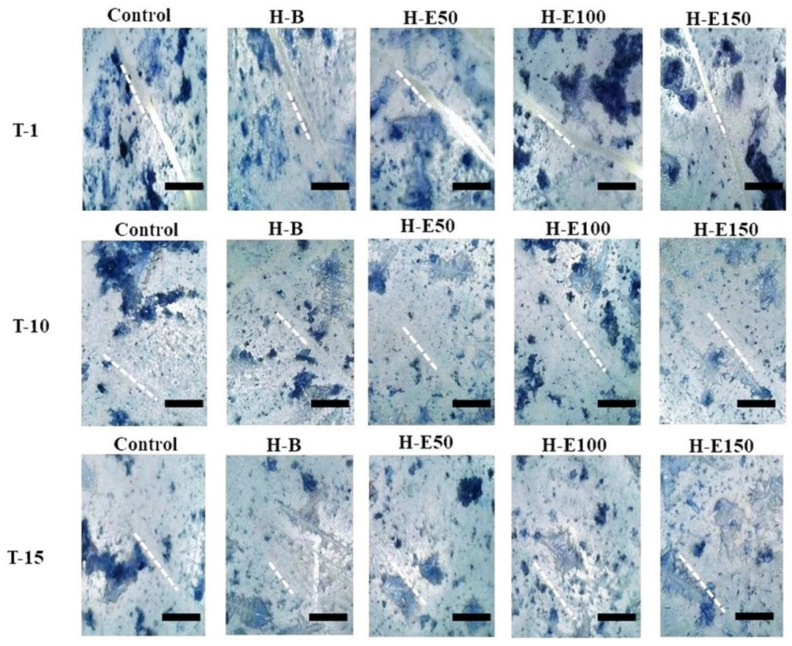
Representative images of the in vitro wound healing assay showing the closure of scratch defects in porcine dermis fibroblasts treated with hydrogel leachates containing varying concentrations (0–21 wt.%) of immobilized *F. cernua* extracts over 15 days. The scale bar is equal to 300 µm.

Finally, the wound healing efficacy of the hydrogels containing immobilized plant extract was assessed by evaluating the migratory and defect-filling capacity of porcine dermis fibroblasts via an in vitro scratch assay. Figure 9 illustrates the wound closure progression at three time points: immediately after the scratch (day 0, T1, initial scratch width ~50 μm), day 10 (T10), and day 15 (T15).

By day 15, complete closure (100%) is observed in all experimental groups. Notably, at day 10, partial closure is evident, with the hydrogel containing 14 wt.% encapsulated extract (H-E100) demonstrating approximately 90% wound closure (Table 1). This performance was statistically superior compared to the extract-free hydrogel (H-B), indicating that the globular morphology induced by this extract concentration within the collagen fibers significantly enhances fibroblast adhesion and migration, thereby promoting the formation of confluent monolayers critical for defect repair. Furthermore, the sustained release profile associated with H-E100, facilitated by the semi-crystalline scaffold architecture and optimized crosslinking density, likely improves the bioavailability of immobilized phenolic compounds, augmenting their biological efficacy. The controlled degradation rate and surface topography of this scaffold create a microenvironment conducive to fibroblast activation and extracellular matrix remodeling.

These biological effects could be attributed to phenolic compounds within the hydrogel matrix, which are known to enhance cell adhesion through interactions with extracellular matrix proteins, such as fibronectin and collagen, that mediate fibroblast attachment [85]. Moreover, these phytochemicals could modulate key intracellular signaling pathways regulating cell proliferation and directed migration [86]. The presence of a phenolic gradient within the scaffold likely acts as a chemotactic cue, guiding fibroblast migration toward the wound site and accelerating tissue repair [87]. Additionally, the potent antioxidant properties of the immobilized phenolics reduce oxidative stress in the wound milieu by scavenging reactive oxygen species (ROS), thereby protecting resident fibroblasts and other regenerative cells from oxidative damage. This antioxidative protection further promotes a favorable environment for efficient wound closure and tissue regeneration [88]. Although ethanol sterilization and extract processing resulted in an approximate 87% reduction in total phenolic content, this value reflects an overall quantification and does not necessarily represent the loss of all biologically relevant phenolic species. Several phenolic compounds identified in the extract, such as caffeic acid, quercetin, apigenin, and myricetin, are chemically stable molecules that can retain their bioactivity even at relatively low concentrations. Furthermore, a fraction of these phenolic species remains immobilized within the collagen-based hydrogel matrix through physical interactions, which may enhance their local biological efficacy by limiting rapid diffusion and degradation and promoting sustained availability at the site of application. Consequently, the observed biological effects are attributed to the controlled release and local activity of the retained phenolic fraction rather than to the total phenolic content initially incorporated. Moreover, the sterilization protocol was applied consistently across all batches, ensuring reproducible phenolic retention and release behavior.

## 3. Conclusions

This work reports the development of bioactive collagen scaffolds encapsulating fermented *F. cernua* extracts, designed to enhance immunomodulation and wound healing. The incorporation of polyphenol-rich extracts (7–21 wt.%) enabled the fabrication of multifunctional hydrogels with tunable physicochemical and biological properties. Increasing the extract content improved crosslinking density (>45%) and water retention capacity (>1400%), while accelerating gelation time—features associated with the progressive integration of extract components into the polymeric network. SEM analysis revealed a fibrillar structure enriched with amorphous globular domains at higher extract concentrations, enhancing thermal stability. Although matrix stiffness decreased with extract loading due to repulsive interactions between extract molecules and the polymeric chains, all hydrogels maintained adequate structural integrity and showed sustained degradation under physiological and skin-mimicking pH conditions. The 14 wt.% extract formulation (H-E100) proved particularly effective, enabling a sustained release of bioactive compounds starting from day 5 under both physiological and acidic (pH 4.5) skin-like conditions. This formulation also significantly promoted fibroblast and monocyte proliferation and metabolic activity, while the 7 wt.% formulation (H-E50) reduced TNF-α secretion, suggesting anti-inflammatory potential. Hemocompatibility was confirmed in all cases, with no hemolytic activity detected. In vitro scratch assays demonstrated up to 90% wound closure within 10 days in fibroblast cultures treated with the H-E100 hydrogel, highlighting its regenerative efficacy. Overall, *F. cernua*-enriched collagen hydrogels exhibit great promise as bioactive scaffolds for sustained phytochemical delivery, offering a multifunctional platform for biomedical applications.

## 4. Materials and Methods

### 4.1. Biological Material

Foliage of *F. cernua* was harvested during summer season in the southeastern region of Saltillo, Coahuila. Following collection, the plant material was dehydrated at 50 °C for 72 h and subsequently stored at ambient temperature until further use. For the fermentation process, a strain of *Aspergillus niger*, originally isolated from *A. lechuguilla* and supplied by the culture collection of the Environmental Biotechnology Department at the Universidad Autónoma de Coahuila (UAdeC) (Saltillo, Mexico), was employed. The fungal strain was preserved and preconditioned following the protocol outlined in reference [89].

### 4.2. Preparation of F. cernua Extract via Solid-State Fermentation

The inoculum was prepared by suspending fungal spores—previously propagated—using a 0.1% (*v*/*v*) Tween 80 solution. The concentration of spores in the suspension was determined with a Neubauer counting chamber (Marienfeld Superior, Lauda-Königshofen, Germany), allowing for the calculation of the precise quantity required for fermentation [90]. For the fermentation process, 15 g of dried *F. cernua* leaves were evenly distributed in aluminum trays (12.5 cm in diameter × 3 cm deep). The solid-state fermentation (SSF) was performed at 30 °C over 48 h, maintaining a relative humidity of 85%. The initial inoculum consisted of 2 × 10^7^ spores per gram of substrate. To maintain appropriate moisture levels during fermentation, a Czapeck-Dox mineral solution adjusted to pH 4.5 was employed. This medium included (g/L): 2.0 NaNO_3_, 1.0 KH_2_PO_4_, 0.25 KCl, and 0.75 MgSO_4_, and was sterilized by autoclaving at 121 psi for 15 min before use. After completing fermentation, 15 mL of a hydroalcoholic solution was added to the fermented biomass and allowed to interact for 5 min. The biomass was then manually pressed to recover the bioactive-rich extract. Finally, the extract was subjected to centrifugation at 3500 rpm for 15 min and subsequently filtered for further analysis [12].

### 4.3. Determination of Total Phenolic Compounds (TPC) and Evaluation of Antioxidant Activity of F. cernua Extracts

The concentration of hydrolyzable phenolic compounds in the *F. cernua* extracts was quantified using the Folin–Ciocalteu colorimetric method. Gallic acid served as the calibration standard, and the phenolic content was expressed as milligrams of gallic acid equivalents per gram of extract (mg GAE/g). Flavonoid content was assessed using the aluminum chloride (AlCl_3_) colorimetric assay, with quercetin as the reference compound; results were reported in milligrams of quercetin equivalents per gram of sample (mg QE/g). The phytochemical profile of the fermented extracts was previously analysed by HPLC-MS (Table 2) [12]. The antioxidant potential of the extracts was examined by employing the DPPH (2,2-diphenyl-1-picrylhydrazyl) and ABTS (2,2′-azino-bis (3-ethylbenzothiazoline-6-sulphonic acid)) radical scavenging assays. In both methods, the antioxidant capacity was expressed as the percentage inhibition of the respective free radicals, following protocols previously described in the literature [91].

### 4.4. Preparation of Collagen Hydrogels with Encapsulated F. cernua Extracts

Type I collagen, enzymatically extracted from porcine dermis (Municipal slaughterhouse, Saltillo, Mexico) and characterized by molecular weights of 220,000 Da and 110,000 Da, was used at a fixed concentration of 6 mg·mL^−1^, following the methodology previously reported by [92]. The crosslinking agent consisted of a waterborne polyurethane synthesized via the reaction between glycerol ethoxylate and hexamethylene diisocyanate (HDI), as described in [92,93]. The fermented extracts obtained in the earlier phase were first processed by rotary evaporation to remove the ethanol content. Subsequent centrifugation and filtration steps were employed to eliminate any remaining spores. To ensure sterility, hydrogen peroxide was added at a concentration of 10 µL per 100 mL of extract, followed by a 10 min exposure to ultraviolet (UV) light under a laminar flow hood. The pH of the sterilized extracts was adjusted to 7.21 before incorporation.

Hydrogel synthesis was carried out by thoroughly mixing the extracts with collagen in varying proportions to achieve final concentrations of 7 wt.%, 14 wt.%, and 21 wt.% (*w*/*w* with respect to collagen) of extracts. From this mixture, 1 mL was dispensed into each well of a 24-well culture plate. The crosslinking step was initiated by adding 30 wt.% waterborne polyurethane (relative to the collagen content), and 200 µL of 10× phosphate-buffered saline (PBS) was incorporated to adjust the reaction pH to 7.4. The entire formulation was mixed uniformly and incubated at 37 °C for 4 h to allow for complete gelation [94]. The specific compositions and formulations of the hydrogel systems containing immobilized *F. cernua* extracts are detailed in Table 3.

### 4.5. Study of the Structure and Properties of Hydrogels

To evaluate the integration of the extracts within the polymeric matrix and possible structural modifications, several analytical techniques were applied.

After complete gelation, the hydrogels were used in their hydrated state for swelling, gelation kinetics, rheological analyses, and biological response. For structural and physicochemical characterization, selected samples were converted into xerogels by controlled drying at room temperature under ambient conditions (25 °C) until constant weight was achieved (48 h). This dehydration step was performed exclusively for analytical purposes and did not alter the intended hydrated application of the materials. Drying was carried out without vacuum or thermal treatment to minimize structural collapse.

#### 4.5.1. Structural and Chemical Characterization

The internal architecture and surface morphology of the xerogels were analyzed using scanning electron microscopy (SEM). Dried hydrogel samples (100 mg) were adhered to carbon-coated aluminum stubs to minimize charge interference and examined under a JEOL JSM-7600F microscope (JEOL Ltd., Tokyo, Japan)to assess porosity and extract dispersion. SEM observations were carried out under high-vacuum conditions using xerogels, as water-free samples are required for imaging. The resulting micrographs were used to qualitatively assess relative morphological features rather than to directly represent the hydrated hydrogel structure.

Fourier-transform infrared (FTIR) spectroscopy was used to detect functional group variations following extract immobilization. Spectra were acquired on a Perkin Elmer Frontier spectrometer (PerkinElmer Inc., Waltham, MA, USA) with a diamond attenuated total reflectance (ATR) accessory. Each sample was scanned 16 times at a resolution of 16 cm^−1^, within the 4000–600 cm^−1^ range.

To assess possible changes in molecular organization and crystallinity, X-ray diffraction (XRD) analysis was performed on xerogel samples using an Anton Paar SAXS-Emc2 system equipped with a Cu Kα source (λ = 1.54 Å) (Anton Paar GmbH, Graz, Austria). The analysis allowed identification of amorphous or crystalline regions and how these were affected by increasing concentrations of encapsulated extracts.

The degree of covalent cross-linking achieved during hydrogel formation was estimated indirectly via the ninhydrin reaction. Hydrogel samples were immersed in 3 mL distilled water and reacted with 1 mL of ninhydrin reagent. After vortexing, the mixtures were incubated at 90 °C for 2 h in a dry bath. Absorbance at 567 nm was measured using a MultiSkan Sky (Thermo Fisher Scientific, Waltham, MA, USA)UV-Vis spectrophotometer. Controls were prepared using soluble collagen, PBS, ninhydrin, distilled water, and extract solutions. All experiments were carried out in triplicate. Crosslinking percentages were calculated by comparing the absorbance of modified versus unmodified collagen.

#### 4.5.2. Physicochemical and Functional Characterization

The polymerization kinetics during gelation were monitored via turbidimetric analysis. A 200 µL aliquot of each precursor solution was transferred into a 96-well microplate, and gelation was initiated at 37 °C within the UV-Vis spectrophotometer chamber. Absorbance at 406 nm was measured every 30 s for 4 h to follow the formation of the polymeric network in real time.

The hydration behavior and water retention capacity of the hydrogels were evaluated gravimetrically. Samples were weighed immediately after crosslinking in their fully hydrated state and subsequently dried at room temperature until constant weight to obtain xerogels. The hydration-related swelling percentage was calculated from the mass difference between hydrated and dry states, reflecting the intrinsic water content and retention capacity of the polymeric network. Swelling measurements correspond to the native swelling behavior of the hydrogels immediately after synthesis, without prior drying. This approach was selected to evaluate the intrinsic water uptake capacity of the freshly formed networks.

Additional swelling experiments were conducted to evaluate the water uptake behavior and hydrolytic stability of the xerogels under different pH conditions. After synthesis, the hydrogels were subjected to controlled dehydration to obtain xerogels, which were subsequently used in the swelling assays. Prior to immersion, the xerogels were gently rinsed with distilled water to remove loosely bound residues and then allowed to equilibrate under ambient conditions.

The xerogels were incubated in aqueous buffer solutions at pH 4.5, 7.4, and 8.5, representing acidic (skin-related), physiological, and mildly alkaline environments associated with infected wounds, respectively. At predetermined time intervals up to 72 h, the samples were removed from the solutions, carefully blotted with filter paper to eliminate excess surface water, and immediately weighed. The swelling behavior was evaluated by monitoring the relative weight increase in the xerogels over time with respect to their initial dry weight.

Thermal stability and degradation profiles were assessed through thermogravimetric analysis (TGA). Xerogel samples were heated from room temperature to 800 °C at a rate of 20 °C/min. Nitrogen was used as the carrier gas up to 600 °C, followed by oxygen from 600 °C to 800 °C, using a Perkin Elmer (PerkinElmer Inc., Waltham, MA, USA) TGA-4000 analyzer.

To explore the viscoelastic behavior of the hydrogels, rheological measurements were performed with an Anton Paar MCR 300 rheometer (Anton Paar GmbH, Graz, Austria). A parallel plate setup (40 mm diameter) was used, and measurements were conducted at 37 °C using a solvent trap to limit water evaporation. A constant strain of 10% was applied within the linear viscoelastic region. The storage modulus (G′), representing the elastic behavior, was recorded for each sample in triplicate.

The fatigue behavior of the hydrogels was evaluated under dynamic cyclic conditions using an orbital incubator set at 37 °C to simulate physiological temperature. Hydrogel samples were placed in the incubator and subjected to continuous mechanical agitation at 350 rpm, generating repetitive deformation cycles. The fatigue response was assessed after 504,000 and 1,008,000 cycles by measuring the permanent deformation of the hydrogels. The deformation (strain) of the hydrogels during the fatigue tests was determined by comparing the sample dimensions before and after cyclic loading. Specifically, the relative change in the characteristic dimension of each hydrogel sample was measured after the predetermined number of cycles and expressed as a percentage with respect to its initial dimension prior to testing. This approach was used to compare the resistance of the different hydrogel formulations to long-term cyclic mechanical stress and to evaluate the effect of *F. cernua* extract incorporation on fatigue performance.

The adhesion behavior of the hydrogels was qualitatively evaluated using an inverted adhesion test. A representative hydrogel formulation (H-E100) was placed in contact with various substrates, including human skin, polypropylene, glass, and cotton fabric. The hydrogel was positioned in an inverted configuration, with the material facing downward against gravity. Adhesion was assessed by visual inspection and photographic documentation, based on the ability of the hydrogel to remain attached to each substrate without detachment under its own weight.

Degradability was examined under conditions simulating physiological environments. Each hydrogel was incubated at 37 °C in a medium containing 1× PBS (pH 4.5, related to the skin) and collagenase (14 U per sample). Mass loss was recorded daily for 30 days. The initial and final weights were compared to calculate the degradation rate over time.

#### 4.5.3. Release Profile Determination of Extract from Hydrogels

To evaluate the release behavior of the encapsulated *F. cernua* extract, hydrogels were immersed in aqueous buffer solutions at pH 4.5 and pH 7.4, simulating skin and physiological conditions, respectively. Each hydrogel sample was carefully placed in individual containers containing 10 mL of the respective buffer solution and maintained at physiological temperature (37 °C) throughout the experiment.

At predetermined time intervals over a period of 15 days, aliquots of 200 µL were withdrawn from each container to monitor the concentration of the released extract in the surrounding medium. During the release experiments, aliquots were withdrawn at predetermined time intervals and replaced with an equal volume of fresh buffer solution in order to maintain sink conditions and a constant concentration gradient throughout the experiment. This approach prevents medium saturation and ensures reliable monitoring of release kinetics. Cumulative release values were calculated by applying the appropriate dilution correction to account for sample replacement at each time point. The presence of released extract in each aliquot was detected by measuring the absorbance using a UV-Vis spectrophotometer at 610 nm. The wavelength of 610 nm was selected because it corresponds to the maximum absorption peak of the released extract under the experimental conditions employed. This absorption maximum reflects the combined contribution of extract constituents and their interactions with the collagen-based network, which can induce bathochromic shifts due to hydrogen bonding, π–π interactions, and complex formation. Additionally, monitoring at 610 nm minimizes spectral interference from collagen and other matrix components, which exhibit significant absorption at lower wavelengths. Therefore, the selected wavelength provides a more selective and reliable assessment of extract release from the hydrogel system. The absorbance readings with respect to time were graphed to know the release profiles of the extracts in this type of hydrogel. All measurements were performed in triplicate to ensure reproducibility. This method allowed for detailed monitoring of the release kinetics and assessment of the influence of pH on the extract’s diffusion from the hydrogel matrix.

### 4.6. Evaluation of Biological Response In Vitro

#### 4.6.1. Animal Cell Cultures

Animal cell cultures were established using a modified procedure based on the method described by Caldera-Villalobos [93]. Human blood was collected from healthy adult volunteers in accordance with a protocol approved by the Ethics Committee of the Faculty of Chemical Sciences, Autonomous University of Coahuila (Protocol No. P-FCQ-H-01-09-21-1). All participants provided written informed consent prior to sample collection. Monocytes were isolated from a human blood sample that was centrifuged at 3000 rpm for 30 min. Following centrifugation, the plasma (supernatant) was discarded, and the remaining cells were rinsed in 1× PBS for 10 min. The washed cells were then transferred to Falcon tubes containing Roswell Park Memorial Institute (RPMI) medium at a concentration of 10.44 g/L, supplemented with 200 µL of penicillin-streptomycin. Cultures were incubated at 37 °C, with medium replacement every 48 h using RPMI medium. Cell density was determined using a Neubauer hemocytometer, and cultures were adjusted to 30,000 cells/mL.

For fibroblast co-culture, samples were collected from porcine dermal and subcutaneous tissues, obtaining approximately 15 g of biological material. The tissue was washed three times with sterile 1× PBS, each wash lasting 10 min, and subsequently rinsed in 100 mL of sterile 1× PBS. From this suspension, 45 mL was transferred into a Falcon tube. To facilitate enzymatic degradation of the extracellular matrix, 5 mL of 1× trypsin, 500 µL of a collagenase solution (14 U), and 10 µL of penicillin-streptomycin were added. The mixture was incubated at 37 °C under agitation for 1 h. After digestion, the suspension was washed and centrifuged for 5 min to collect the cellular pellet. Recovered cells were resuspended in sterile culture flasks containing 45 mL of a solution composed of 600 mg of DMEM powder, 500 mL of simulated body fluid (SBF), and 200 µL of penicillin-streptomycin. Cultures were maintained at 37 °C and quantified using a Neubauer chamber until a density of 30,000 cells/mL was achieved.

#### 4.6.2. Hemolysis Test

To evaluate the hemocompatibility of the selected samples, a hemolysis assay was conducted in accordance with ISO 10993-4 guidelines and institutional ethical protocols mentioned above. Peripheral human blood was collected and centrifuged at 3000 rpm for 5 min to separate erythrocytes. The red blood cells were washed three times using 5 mL of Alsever’s solution (Sigma-Aldrich, St. Louis, MO, USA) to remove plasma and debris. Subsequently, 100 µL of the cleaned erythrocytes were diluted into 10 mL of fresh Alsever’s solution to prepare the working suspension. For the assay, 100 µL (100 mg) of the hydrogel sample was combined with 1288 µL of Alsever’s solution and 112 µL of the erythrocyte suspension. The mixture was incubated at 37 °C for 1 h. After incubation, the samples were centrifuged again at 3000 rpm for 5 min. Hemoglobin release, indicative of erythrocyte membrane disruption, was quantified by measuring the absorbance of the supernatant at 415 nm using a Multiskan Sky Reader spectrophotometer (Thermo Fisher Scientific, Waltham, MA, USA) [95]. The extent of hemolysis was quantified as a percentage, calculated according to Equation (1):

(1) $$\%\;Hemolysis=\frac{ASample-Acontrol(-)}{Acontrol(+)\;-\;Acontrol(-)}\times 100.$$

In this context, the positive control absorbance (Acontrol(+)) corresponds to the value obtained using distilled water, while the negative control absorbance (Acontrol(−)) corresponds to Alsever’s solution. These values were compared against the absorbance of the hydrogel samples (ASample) to determine hemolytic activity.

#### 4.6.3. Assessment of Cell Viability

Initially, 100 µL of hydrogel formulations containing 30,000 cells per well—comprising human monocytes and porcine dermal fibroblasts—were seeded into cell culture plates. The cultures were then incubated at 37 °C for time points of 24 and 48 h. Sterile 1× PBS was used as the positive control. To assess cell viability, 10 µL of MTT reagent (1 wt./v.%) (Sigma-Aldrich, St. Louis, MO, USA)was added to each well, followed by a 3 h incubation at 37 °C to allow for formazan formation. Subsequently, 100 µL of isopropanol (propan-2-ol) was introduced to solubilize the formazan crystals. An aliquot of 30 µL from each well was then diluted with 170 µL of isopropanol, and the absorbance was recorded at 570 nm using a spectrophotometer [96,97]. Cell viability was calculated according to Equation (2):

(2) $$\%\;Cell\;viability=\;\frac{ASample}{AControl}\;\times 100,$$

where ASample and Acontrol refer to the absorbance values of the tested hydrogel and the 1× PBS control, respectively. According to ISO 10993-5, cell viability values below 60% are considered indicative of cytotoxic effects, reflecting impaired cellular metabolic activity.

#### 4.6.4. Evaluation of Cell Proliferation

A volume of 1 mL from each cell type was co-cultured with 1 mL of the corresponding hydrogel formulation and incubated for 48 h at 37 °C. After incubation, human monocytes were stained with fluorescein diacetate (FDA) (Sigma-Aldrich, St. Louis, MO, USA), while porcine dermal fibroblasts were labeled with rhodamine B (Sigma-Aldrich, St. Louis, MO, USA). FDA is a non-fluorescent compound that passively diffuses into viable cells and is hydrolyzed by intracellular esterases to release fluorescein, which emits green fluorescence and is retained in cells with intact membranes, serving as a marker of viability and metabolic activity [70]. Rhodamine B, a lipophilic dye, accumulates in the cytoplasm of live cells with intact membrane potential and emits red fluorescence upon excitation, allowing the identification of viable fibroblasts [72].

To ensure adequate incorporation into the membranes of metabolically active cells, the cultures with fluorescent markers were incubated for an additional 4 h. Subsequently, the stained cells were centrifuged at 3000 rpm for 15 min, and the resulting pellets were mounted on glass slides for observation under fluorescence EPI microscopy. Cell proliferation was visualized using a VELAB VE-146YT microscope equipped with a 40× objective (VELAB Co., McAllen, TX, USA). Fluorescence excitation was performed using a blue laser (for fluorescein) and a green laser (for rhodamine B) [95], in accordance with ISO 10993-6 for the evaluation of local tissue effects following implantation.

#### 4.6.5. Scratch Assay for in Vitro Testing of Cell Migration

The cell migration of hydrogels containing immobilized extract was assessed using an in vitro scratch assay, following guidelines consistent with ISO 10993-5 for cytotoxicity and cell migration evaluation. Porcine dermal fibroblasts were seeded at a density of 2 × 10^5^ cells per well by applying 100 µL of cell suspension onto sterile coverslips. The cultures were incubated at 37 °C in a humidified atmosphere for 48 h to allow the formation of a confluent monolayer. After monolayer establishment, 20 µL of each hydrogel sample and 50 µL of trypan blue solution were added to assess cell viability and to visualize dead cells. A linear scratch (“wound”) was carefully created in the center of each monolayer using a sterile spatula, generating a cell-free area that mimics a wound site. This scratch disrupts the monolayer, allowing observation of cell migration and proliferation as cells move to close the gap over time. The process of wound closure was monitored and documented through periodic photographic imaging using a portable LCD digital microscope (model G1000) at predetermined intervals [98]. The scratch assay serves as a simple and reproducible in vitro model to evaluate the regenerative potential of biomaterials, by measuring the ability of fibroblasts to migrate and proliferate to repopulate the wounded area. Cell migration and wound closure kinetics are key indicators of the biomaterial’s effect on tissue repair and healing [99].

#### 4.6.6. Effect of Hydrogels on Monocyte Cytokine Release

The impact of hydrogel composition on cell signaling pathways was evaluated by measuring the secretion of key cytokines associated with the inflammatory response during wound healing. Enzyme-linked immunosorbent assays (ELISA) (Invitrogen, Thermo Fisher Scientific, Waltham, MA, USA) were conducted according to the manufacturer’s protocol to quantify tumor necrosis factor-alpha (TNF-α) levels in human monocytes cultured on hydrogels with immobilized extracts for 48 h [95]. TNF-α is a pro-inflammatory cytokine that plays a crucial role in the early stages of wound healing by regulating immune cell recruitment, activating macrophages, and influencing tissue remodeling. Elevated or prolonged TNF-α expression can indicate inflammation, which must be carefully balanced to avoid chronic wounds or excessive scarring. Therefore, assessing TNF-α secretion provides valuable insight into how hydrogels modulate the inflammatory microenvironment to support tissue repair [100,101].

### 4.7. Statistical Analysis

All experiments were performed in triplicate, and the results are reported as mean values with the corresponding standard deviations. The results were considered statistically significant at a confidence level of *p* < 0.05. The data are presented as the mean ± standard deviation (SD). Comparisons among groups were conducted using one-way analysis of variance (ANOVA). To determine which groups differed significantly, the Least Significant Difference (LSD) test was applied, with significance set at *p* < 0.05.

## Figures and Tables

**Table 1:** Hemocompatibility, TNF-α cytokine secretion by monocytes, and in vitro wound closure by fibroblasts in response to hydrogels containing different concentrations of immobilized *F. cernua* extracts.

Hydrogel	Hemolysis (%)	TNF-α (pg/mL)	Wound Contraction (%) (at Day 10)
H-B	0.0 ± 0.01	0.0 ± 22.63	76.0 ± 5.37
H-E50	1.4 ± 0.01	7.2 ± 15.25	82.0 ± 12.03
H-E100	0.0 ± 0.00	14.2 ± 12.73	90.0 ± 8.34 *
H-E150	0.0 ± 0.00	34.2 ± 14.04 *	78.0 ± 4.92

Values are expressed as mean ± standard deviation, * statistically significant differences (*p* < 0.05) are indicated between extract-containing hydrogels and the extract-free control (H-B).

**Table 2:** HPLC-MS analysis of *F. cernua* fermented extracts (Adapted from reference [12]).

Compound	Retention Time	*m*/*z*	Family
Myricetin	5.428	316.9	Flavonols
Apigenin 6,8-di-C-glucoside	21.038	592.8	Flavones
Apigenin galactoside-arabinoside	23.088	562.8	Flavones
Protocatechuic acid 4-O-glucoside	35.786	314.9	Hydroxybenzoic acids
Hispidulin	37.809	298.9	Methoxyflavones
3,7-Dimethylquercetin	40.182	328.9	Methoxyflavonols
Cirsimaritin	42.309	312.9	Methoxyflavones
Eupatorin	44.034	342.8	Methoxyflavones
Pebrellin	45.916	342.8	Methoxyflavones

**Table 3:** Collagen hydrogel compositions with encapsulated bioactive extracts from *F. cernua*.

Formulation	Collagen (mg)	Polyurethane Crosslinker (mg)	*F. cernua* Extract (mg)	*F. cernua* Extract (wt.%)
H-B	6.0	1.8	0.0	0.0
H-E50	6.0	1.8	0.4	7.0
H-E100	6.0	1.8	0.8	14.0
H-E150	6.0	1.8	1.2	21.0

Note: Extract concentrations higher than 21 wt.% inhibit the crosslinking reaction, preventing proper hydrogel formation.

## Data Availability

Data will be made available upon reasonable request.
